# Whole-Genome Investigation of Zoonotic Transmission of Livestock-Associated Methicillin-Resistant *Staphylococcus aureus* Clonal Complex 398 Isolated from Pigs and Humans in Thailand

**DOI:** 10.3390/antibiotics12121745

**Published:** 2023-12-16

**Authors:** Pawarut Narongpun, Pattrarat Chanchaithong, Junya Yamagishi, Jeewan Thapa, Chie Nakajima, Yasuhiko Suzuki

**Affiliations:** 1 Division of Bioresources, Hokkaido University International Institute for Zoonosis Control, Sapporo 001-0020, Japan; npawarut@czc.hokudai.ac.jp (P.N.); jeewan@czc.hokudai.ac.jp (J.T.); 2 Department of Veterinary Microbiology, Faculty of Veterinary Science, Chulalongkorn University, Bangkok 10330, Thailand; pattrarat.c@chula.ac.th; 3 Division of Collaboration and Education, Hokkaido University International Institute for Zoonosis Control, Sapporo 001-0020, Japan; junya@czc.hokudai.ac.jp; 4 International Collaboration Unit, Hokkaido University International Institute for Zoonosis Control, Sapporo 001-0020, Japan; 5 Institute for Vaccine Research and Development, Hokkaido University, Sapporo 001-0020, Japan

**Keywords:** livestock-associated methicillin-resistant *Staphylococcus aureus*, LA-MRSA, antimicrobial resistance, whole-genome sequencing, WGS, pigs, workers, occupational diseases, Thailand

## Abstract

Livestock-associated methicillin-resistant *Staphylococcus aureus* (LA-MRSA) has been widespread globally in pigs and humans for decades. Nasal colonization of LA-MRSA is regarded as an occupational hazard to people who are regularly involved in livestock production. Our previous study suggested pig-to-human transmission caused by LA-MRSA clonal complex (CC) 398, using traditional molecular typing methods. Instead, this study aimed to investigate the zoonotic transmission of LA-MRSA CC398 using whole genome sequencing (WGS) technologies. A total of 63 LA-MRSA isolates were identified and characterized in Thailand. Further, the 16 representatives of LA-MRSA CC9 and CC398, including porcine and worker isolates, were subjected to WGS on the Illumina Miseq platform. Core-genome single nucleotide polymorphism (SNP)-based analyses verify the zoonotic transmission caused by LA-MRSA CC398 in two farms. WGS-based characterization suggests the emergence of a novel staphylococcal cassette chromosome (SCC) *mec* type, consisting of multiple cassette chromosome recombinase (*ccr*) gene complexes via genetic recombination. Additionally, the WGS analyses revealed putative multi-resistant plasmids and several cross-resistance genes, conferring resistance against drugs of last resort used in humans such as quinupristin/dalfopristin and linezolid. Significantly, LA-MRSA isolates, in this study, harbored multiple virulence genes that may become a serious threat to an immunosuppressive population, particularly for persons who are in close contact with LA-MRSA carriers.

## 1. Introduction

Over the past two decades, pigs have been regarded as crucial reservoirs of livestock-associated *Staphylococcus aureus* (LA-MRSA). Pig-associated MRSA can be occupationally transmitted to individuals who are frequently in close contact with colonized animals, for example, husbandry workers, veterinarians, and slaughterhouse workers [1,2,3]. Eventually, these groups of people may become asymptomatic carriers of LA-MRSA. Nasal LA-MRSA carriage in humans is associated with a greater risk of developing infections; moreover, carriers can potentially transfer LA-MRSA from farms to their household members, although it has been studied that human-to-human transmission caused by LA-MRSA transmission rarely occurs [4,5,6,7]. To date, it is believed that LA-MRSA tends to be less virulent than the other two strains in healthcare settings and communities, hospital-associated MRSA (HA-MRSA) and community-associated MRSA (CA-MRSA) [8,9]. As previously reported, for infections in healthy hosts, LA-MRSA generally develops mild or local infections; however, severe infections, occasionally leading to death, can be presented in fragile population such as children, the elderly, and patients with immunosuppression or chronic diseases [10,11,12].

At present, LA-MRSA has spread widely in swine farming and pork industrial production, and it has become a growing threat to public health worldwide [13]. In North America and Europe, the most prevalent LA-MRSA lineage belongs to clonal complex (CC) 398. On the other hand, most of the pig population in Asian countries has been successfully occupied by CC9 for years [13]; however, recently, the incidence rate of LA-MRSA CC398 among pig herds has increased dramatically to 87.5% and 96.3% in Thailand and China, respectively [2,14]. According to our previous study in Thailand, conventional molecular typing methods exhibited that LA-MRSA isolates from swine workers and their pigs possessed identical molecular characteristics, and they were also phenotypically resistant to the same antimicrobial agents [2]. These observations suggested inter-species transmission caused by CC398; however, a study exploring higher genetic relatedness among LA-MRSA from both species has never been conducted in Thailand. Currently, several molecular typing methods are used to approach epidemiological studies and outbreak investigations of LA-MRSA, such as staphylococcal cassette chromosome (SCC) *mec* typing, *spa* typing, pulsed-field gel electrophoresis (PFGE), and multi-locus sequence typing (MLST). These typing methods, however, present some limitations. Whole genome sequencing (WGS) is another alternative technique that allows us to access greater resolution and DNA sequence dissimilarities at the nucleotide level. Due to its several benefits, this study aimed to use WGS approaches to illustrate the zoonotic transmission of LA-MRSA CC398 between pigs and farm workers in the central region of Thailand and to characterize their genomic features using public international databases.

## 2. Results

### 2.1. WGS-Based Characteristics

The most prevalent genotype (sequence type-SCC*mec* type-*spa* type) was ST398-SCC*mec*V-t034 (9/16), followed by ST398-SCC*mec* composite island (CI)-t034 (4/16), ST9-SCC*mec*IX-t337 (2/16), and ST4576-SCC*mec*IX-t337 (1/16) (Table 1). The type V SCC*mec* element of the ST398-t034 strain contained a class C2 *mec* gene complex with a type 5 cassette chromosome recombinase (*ccr*) gene complex (*ccrC1*). The SCC*mec* type IX carried by the members of CC9, ST9-t337, and ST4576-t337 clones, consisted of the class C2 *mec* gene complex and a type 1 *ccr* gene complex (*ccrA1B1*). Interestingly, the composite SCC*mec* elements identified in the 4 ST398-t034 isolates were organized by a combination of multiple *ccr* gene complexes, *ccrA1B1* and *ccrC1*, and the *mec* class C2 complex.

### 2.2. Core-Genome Single Nucleotide Polymorphism (SNP)-Based Analyses and Transmission of LA-MRSA CC398

**Figure 1:**
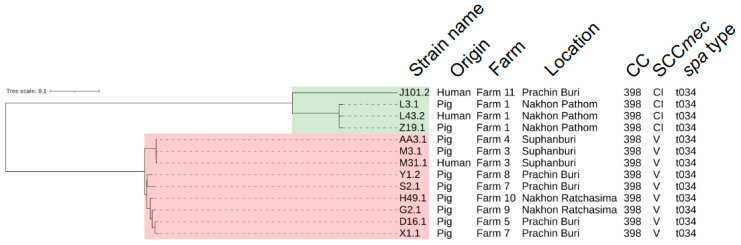
Phylogenetic analysis of the 13 LA-MRSA CC398 isolates based on core genome SNPs. The evolutionary tree was reconstrued using maximum likelihood inference and a bootstrap value of 1000 repetitions. The LA-MRSA isolates of CC398, derived from live pigs (*n* = 10) and healthy swine workers (*n* = 3), diverged into two monophyletic groups, namely, Clade I (green) and Clade II (red). Importantly, the phylogenetic tree depicted two possible events of zoonotic transmission in Farm 1 and Farm 3.

Figure 1 presents the phylogenetic tree based on single nucleotide polymorphisms (SNPs) in the core genome of the 13 LA-MRSA CC398 isolates. The isolates were divided into two distinct phylogenetic clades, namely, Clade I and Clade II. Almost all internal nodes had bootstrap support of 90% or greater. Clade I was occupied by the 4 LA-MRSA isolates carrying the composite SCC*mec* elements from Farm 1 and Farm 11. Two porcine isolates and one human isolate from Farm 1 apparently were clustered in the same sub-clade, elucidating a high degree of genome relatedness among them; however, one human isolate from Farm 11 was distantly related to those from Farm 1. Clade II was completely clustered by all LA-MRSA isolates carrying the SCC*mec* type V. The LA-MRSA isolates from Farm 3 and Farm 4, located in Suphanburi, were placed into the same sub-clade showing a geographical specificity (Supplementary Materials Figure S1). Like Farm 1, one porcine isolate from Farm 3 was clustered tightly with a human isolate obtained from the same location. The other six porcine isolates in Clade II, obtained from five different farms in Prachin Buri and Nakhon Ratchasima, resided together in a discrete sub-clade.

To verify all transmission events detected in this study, a pairwise SNP distance matrix made from the core genome alignment was investigated (Supplementary Materials Table S1). In Clade I, the pairwise SNP distances between the two porcine strains L3.1 and Z19.1 from Farm 1 were 13-core genome SNPs. These porcine strains were even closer to the human strain L43.2 from the same farm with 11 and 10 SNPs, respectively. On top of that, in Clade II, the porcine strain M3.1 from Farm 3 was only three SNPs distant from the human strain M31.1 from the same farm. This indicates that both isolates were genomically related and originated from the same root. For Farm 4, the porcine strain AA3.1 was separated from both isolates from Farm 3 (the strains M3.1 and M31.1) by 2 and 3 SNPs, respectively. The remaining porcine strains in this clade, collected from Prachin Buri and Nakhon Ratchasima, shared a common ancestor with a different number of SNP distances ranging from 14 to 38 SNPs.

### 2.3. Antimicrobial Resistance-Associated Genes and Stress Genes Profile

In total, we identified 32 antimicrobial resistance-associated genes, mediating resistance against 14 antimicrobial groups: aminoglycosides (*n* = 6), beta-lactams (*n* = 3), fluoroquinolones (*n* = 2), fosfomycin (*n* = 3), glycopeptide antibiotics (*n* = 1), lincosamides (*n* = 1), lincosamides-pleuromutilins-streptogramin A compounds (PLS_A_) (*n* = 3), macrolide-lincosamide-streptogramin B compounds (MLS_B_) (*n* = 3), phenicols (*n* = 2), phenicols-oxazolidinones-lincosamides-pleuromutilins-streptogramin A compounds (PhLOPS_A_) (*n* = 1), rifampicin (*n* = 1), tetracyclines (*n* = 4), trimethoprim (*n* = 1), and multidrug efflux MATE (multidrug and toxic compound extrusion) transporter (*n* = 1) (Table 2). It is noteworthy that PLS_A_, MLS_B_, and PhLOPS_A_ phenotypes are related to cross-resistance, which refers to an ability of resistance to several classes of antimicrobials by a single mechanism [15,16].

It is revealed that all LA-MRSA isolates could be defined as multidrug-resistant (MDR) MRSA due to resistance against at least three classes of antimicrobials. In addition, all of them tested positive for *mecA*, S84L mutation in *gyrA*, *tet*(38), *tet*(M), *dfrG*, and *mepA*. In contrast, the vancomycin resistance gene *vanA* and the mutation of *rpoB* gene were not presented in any isolates.

The *in-silico* detection of antimicrobial resistance gene patterns among LA-MRSA isolates within each genotype were highly similar. Within the CC398 subpopulation, the ST398-SCC*mec*CI-t034 genotype did not exhibit a significant difference with the ST398-SCC*mec*V-t034 genotype, except for *aac(6′)-Ie/aph(2″)-Ia,* mutations in *parC* and *erm* genes; however, it should be mentioned that, when comparing the patterns of gene carriage and mutations between the CC9-t337 and CC398-t034 subpopulations, we observed the distribution of some resistance genes or mutations that was evidently associated with a particular subpopulation. As shown in Table 2, the streptomycin resistance gene *str* was localized only in the CC9-t337 clone, but the other three aminoglycosides-related genes, *ant(6)-Ia*, *ant(9)-Ia*, and *spw*, were specifically found in the CC398-t034 clone. While the CC9-t337 strains mediated resistance to fosfomycin by encoding protein FosB, all LA-MRSA with the CC398-t034 genotype conferred resistance through mutations of *glpT* A100V/F31I and *murA* D278E/E291D [17,18]. For the PLS_A_ resistance phenotype, the CC398-t034 clone expressed resistance through the encoding *lsa*(E) gene; however, the CC9-t337 clone harbored *vga*(A) genes conferring resistance against PLS_A_ antibiotics. It is important to highlight that some resistance phenotypes were limited to either the CC9-t337 or CC398-t034 subpopulation. The *lnu*(B) gene, involving resistance to lincosamides, was present in all isolates with the CC398-t034 genotype. On the one hand, the strain Q10.1 from the CC9-t337 clone possessed the *catA* gene, showing resistance to phenicols. Also, the strain BA3.1 carried the phenicols resistance gene *fexA* as well as the *cfr* gene exhibiting resistance to PhLOPS_A_.

We also examined the absence/presence of antimicrobial resistance genes distributed among LA-MRSA isolates involved in zoonotic transmission. Undoubtedly, LA-MRSA isolates from swine workers showed an almost 100% identity of antimicrobial resistance gene carriage with those from pigs isolated from the same farm origin.

Besides screening for antimicrobial resistance genes, stress genes, including biocide and metal resistance genes, were also determined. A total of five stress genes, including *arsB*, *arsC*, *lmrS*, *mco*, and *qacG*, were detected (Supplementary Materials Table S2). The most prevalent genes were *lmrS* and *mco*, carried by all LA-MRSA isolates. The arsenic resistance-related genes, *arsB* and *arsC,* were significantly associated with all LA-MRSA belonging to ST398-SCC*mec*CI-t034 and CC9-SCC*mec*IX-t337 genotypes; however, *qacG* resulted positive for the three strains Q10.1, Y1.3, and S2.1 from both CC9-t337 and CC398-t034 subpopulations.

### 2.4. Virulence Gene Repertoire

To assess whether pig-associated LA-MRSA would become a serious issue in human medicine, a total of 76 virulence genes were analyzed and further classified into 5 categories, according to their functions: adherence (*n* = 13), exoenzymes (*n* = 10), host immune evasion (*n* = 16), iron uptake and metabolism (*n* = 8), and toxins and type IV secretion systems (*n* = 29) (Table 3) [19]. Most of the virulence genes were distributed homogeneously in all subpopulations, except for some genes that were restrictedly occupied by either the CC9-t337 or CC398-t034 subpopulation. For example, the collagen adhesion gene (*cna*) and coagulase gene (*coa*) were exclusively found in the CC398-t034 strain. Conversely, *map*, *sdrD*, *aur*, *cap8F*, *essC, esxB*, *esxC*, staphylococcal enterotoxin (SE) genes *sei*, *sem*, *sen*, *seo*, *seu* and *sey*, and staphylococcal enterotoxins-like toxin (SEL) genes *sel27*, *sel28*, and *selX* were specifically found in the CC9-t337 strains; however, *sel26* was the only SEL gene widespread in all isolates.

Notably, nine adherence genes (*clfA*, *clfB*, *ebp*, *icaA*, *icaB*, *icaC*, *icaD*, *icaR*, and *sdrE*) and seven genes encoding the iron-regulated surface determinant protein (Isd) A-G (*isdA*, *isdB*, *isdC*, *isdD*, *isdE*, *isdF*, and *isdG*) resulted positive for all LA-MRSA isolates. The following virulence genes were, however, totally absent in all isolates: *sak*, *scn*, *chp*, *lukF-PV*, *lukS-PV*, *sea*, *sep*, and *tsst-1* genes.

### 2.5. Mobile Genetic Elements

MobileElementFinder discovered 10 plasmid replicon sequences with diverse combinations of carriage. The most widespread plasmid replicon belongs to types rep7a and repUS43 (16/16), followed by rep19b (9/16), and rep10 and rep21 (8/16). Furthermore, a total of four transposon elements, including Tn*551* (4/16), Tn*554* (4/16), Tn*558* (1/16), and Tn*6009* (9/16), were identified in CC398-t034 isolates, as demonstrated in Table 4.

The plasmid replicon repUS43 carried by different strains was obviously linked to various genetic determinants. It was shown to be associated with *mecA* and *tet*(M) genes in the CC9-t337 subpopulation, whereas the ST398-SCC*mec*CI-t034 and ST398-SCC*mec*V-t034 strains carried this plasmid coupled with the *tet*(M) gene and transposon Tn*6009*, respectively. Similarly, the plasmid replicon rep7a carrying *tet*(K) was predominately present in all CC398-t034 isolates, except for the strain M3.1. On the other hand, the rep7a carrying *str* was detected in all CC9-t337 isolates, one of which (the strain Q10.1) also harbored the chloramphenicol resistance gene *cat*(pC221) on this plasmid. Among the CC398-t034 isolates, both Tn*551* and Tn*554* were carried by all isolates with the composite SCC*mec* element. The *erm*(A) gene, together with *ant(9)-la*, was located on the transposon Tn*554*, but the *erm*(B) gene resided on the transposon Tn*551*. In contrast, the rep7a plasmid associated with the *erm*(C) gene was mainly restricted to the 8 CC398-t034 with the SCC*mec* type V.

Importantly, it is worth nothing that we found three interesting carriages of antimicrobial resistance genes with mobile genetic elements in two isolates. The resistance genes *aadD*, *erm*(B), and *tet*(L) were co-localized on the plasmid sequence of the strain BA3.1. On top of that, this strain also tested positive for the transposon Tn*558* carrying *fexA*. Another multi-resistance gene cluster resided on the rep22 belonging to the strain J101.2. It contained two aminoglycosides resistance genes, *aadD* and *ant(6)-la*, the *blaZ* gene, the lincosamides resistance gene *lnu*(B), and the *Isa*(E) encoding PLS_A_ phenotype.

Apart from the plasmid replicons and transposons, other mobile genetic elements were also determined. Within the CC398-t034 subpopulation, the insertion sequence IS*256* was present in only one isolate, the strain J101.2. The IS*Sau8* were predictably harbored by the following three strains: L43.2, D16.1, and X1.1; however, we found no association between these insertion sequences and antimicrobial resistance genes.

## 3. Discussion

This study, to the best of our knowledge, is the first documentation of a whole-genome investigation of zoonotic transmission caused by LA-MRSA CC398 in Thailand. The nasal carriage of LA-MRSA among pigs and farm workers, suggesting the potential of human colonization and zoonotic transmission, has been previously defined by several reports in different parts of Thailand [1,2,20,21]. These studies used various typing methods based on molecular characterization, for example, classical MLST and SCC*mec* typing. Instead, in this study, WGS approaches were used for the genotypic characterization of LA-MRSA. Overall, the results of WGS-based characterization are nearly in accordance with those of molecular characterization in our previous study [2]. At that time, one porcine strain Z19.1 was typed as SCC*mec* non-typeable (NT) using multiplex PCR assays. Additionally, the PCR-based SCC*mec* typing could not specify an allotype of *ccrC* gene complex; however, these uncertainties were clarified by the WGS analyses in our present study. Together, these findings indicate a higher discriminatory power of WGS beyond other general typing approaches.

The occurrence of the SCC*mec*Cl in this study implies an empirical impact on animal movement through international trading. As seen in Table 1, the composite SCC*mec* elements of the 4 CC398-t034 isolates contained both *ccrA1B1* and *ccrC1* gene complexes, which were detected in CC9-SCC*mec*IX-t337 and CC398-SCC*mec*V-t034 subpopulations, respectively; moreover, it should be noted that LA-MRSA CC398 with the type V SCC*mec* element detected in several Asian countries seems to be strongly associated with the international pig trade. To exemplify this, several LA-MRSA CC398 harboring SCC*mec* type V were detected in imported pigs from two different countries during the quarantine period in Japan [22]. On top of that, the CC398-SCC*mec*V-t034 genotype in pigs was documented by a national survey in South Korea. That country has reportedly imported breeding pigs from Canada, Denmark, and the U.S. [23]. More importantly, one previous study demonstrated that six LA-MRSA isolates with the CC398-SCC*mec*V-t034 genotype, from retail pork and a slaughtered pig in the central region of Thailand, were closely related to Danish LA-MRSA, showing the same characteristics [24]. That study also mentioned that the import of live pigs from Denmark to Thailand would constitute the introduction of the LA-MRSA CC398 strain into the Thai pig population. All things considered, it can be hypothesized that LA-MRSA CC398-t034 with SCC*mec*V had been introduced into Thailand due to the cross-border movements of pigs. Subsequently, it would acquire the additional gene complex (*ccrA1B1*) from the domestic strain (CC9-SCC*mec*IX-t337) via genetic recombination, leading to the emergence of a unique composite of the SCC*mec* element [25]. 

In recent years, the carriage of multiple *ccr* gene complexes has been presented by a Chinese LA-MRSA ST9 from clinical and porcine isolates [26]. In addition, another study from China has reported the presence of coagulase-negative staphylococci (CoNS) carrying multiple SCC*mec* elements [27]. It demonstrated that the isolates with multiple SCC*mec* had a more stable capability to continue *mecA* gene transcription involved in cell wall synthesis. As a result, these isolates did not lose Gram positivity under antibiotic exposure when compared to the ones with a single SCC*mec* element [27]; however, we did not perform any phenotypic analyses regarding multiple SCC*mec* elements.

We elucidate and verify zoonotic transmission caused by LA-MRSA CC398 in two farms using core-genome SNP-based analyses. The phylogenetic tree reconstruction illustrated a high genome similarity between porcine and human isolates. Genomic characteristics support the phylogenetic tree as well as imply that the origin of human LA-MRSA CC398 would be pigs. Ultimately, the pairwise SNP analysis does not only confirm pig-to-human transmission; it also indicates evolutionary changes in the core genome of LA-MRSA CC398 after zoonotic spillover and colonization on human nares. One study in Denmark also used WGS to investigate the zoonotic transmission of LA-MRSA between livestock and farmers. A phylogenetic tree based on core genome SNPs revealed that animal isolates differed from Danish worker isolates by only 3–5 SNPs [28]. The small number of pairwise SNPs is very similar to those in the present study.

Additionally, phylogenetic analysis clearly revealed two distinct clades of LA-MRSA CC398 with their own specific SCC*mec* types. LA-MRSA isolates from the same province or their neighboring province were phylogenetically grouped into the same clade or sub-clade. It is exemplified by the six porcine isolates from Prachin Buri and Nakhon Ratchasima. Nakhon Ratchasima shares its provincial borders with Prachin Buri (Supplementary Materials Figure S1). Unsurprisingly, all porcine isolates from these two provinces in Clade II resided together in the same sub-clade; therefore, it can be stated that these clues speculate as to local transmission events of pig-associated LA-MRSA within the same province or between provinces.

Like the phenotypic susceptibility testing described earlier [2], the WGS analyses exhibited that all LA-MRSA isolates in the present study were identified as MDR as well as harboring several cross-resistance genes. The overall distribution of antimicrobial resistance genes suggests the genetic homogeneity of LA-MRSA in each subpopulation. In other words, the isolates sharing the same genotypic characteristics have a highly similar pattern of antimicrobial resistance gene carriage, although they were collected from different locations or hosts. In addition, LA-MRSA CC9 and CC398 possess different resistance mechanisms against a particular antimicrobial group such as a resistance mechanism against fosfomycin or PLS_A_. It is also important to emphasize that several cross-resistance genes carried by our LA-MRSA isolates do not only confer resistance against antimicrobial agents widely used in livestock, but also antibiotics of last resort in human medicine such as quinupristin/dalfopristin and linezolid; however, analysis of antimicrobial resistance gene carriage using WGS in LA-MRSA CC398 has been explored by a study collecting porcine specimens from a province in central Thailand [24,29], and it is consistent with the results of our study.

Although the *lmrS* gene encoding a multidrug efflux pump is categorized as a stress gene according to the AMRFinderPlus database, it has been proved that this gene is able to implicate in resistance to linezolid, aminoglycosides, macrolides, and phenicols, which is relatively similar to the PhLOPS_A_ phenotype expressed by the *cfr* gene [30]; hence, it can be assumed that all LA-MRSA isolates in this study are highly likely to potentiate resistance against oxazolidinones via the lincomycin resistance protein of *Staphylococcus aureus* (LmrS). Three LA-MRSA strains could develop quaternary ammonium compounds (QACs) resistance due to harboring the *qacG* gene. QAC-based disinfectants are commonly used in livestock farms to chemically kill microorganisms on non-living surfaces; therefore, this gene would probably protect LA-MRSA from commercially available disinfectants promoting bacterial persistence in farm environment.

In this study, WGS approaches also disclosed an abundance and diversity of virulence determinants imposed by LA-MRSA isolates. As expected, toxic shock syndrome, the toxin-1 (TSST-1)-encoding gene (*tsst-1*), Panton–Valentine leucocidin (PVL)-encoding genes (*lukF-PV* and *lukS-PV*), as well as the immune evasion gene cluster (IEC), including *sak*, *scn*, *chp*, *sea*, and *sep*, were absent in all LA-MRSA isolates because they were rarely detected in livestock-derived isolates [31,32,33,34]. In addition, they are well known as crucial virulence determinants promoting the pathogenesis or severity of MRSA infections in humans. Furthermore, only a few SE and SEL genes were found in our LA-MRSA isolates.

There are, however, multiple genes which were carried by our LA-MRSA isolates, including *clfA-B*, *cna*, *icaA-D*, *icaR*, *isdA-G*, and *sdrC-E*. Specifically, the *clfB* genes encoding clumping factors B (ClfB) has been determined to play an important role in nasal colonization in humans and experimental animals [35,36,37]. It could bind to the upper layer of epithelial cells in anterior nares by a ligand-receptor interaction with loricrin and was able to interact with cytokeratin 10 (CK10) expressed on skin epithelium. Further, it has been proved to have an effect on skin and soft tissue infection (SSTI) during the early stage of pathogenesis [38,39]. Seven strains had the *cna* gene, which is responsible for bacterial adherence to collagen in host tissue. With this function, the collagen-binding protein could influence staphylococcal infection in collagen-rich tissues such as heart, joints, and bones, and the cornea [40,41,42,43]. The *ica* operon, consisting of *icaA-D* genes and its negative regulator gene *icaR*, is involved in biofilm production. The slime formation does not only protect bacterial communities against host immunity, but also mediates the release of dispersed cells to new sites of infection and avoids penetration of antimicrobial agents through the staphylococcal biofilm [44,45]. Besides being involved in heme-iron acquisition systems, the IsdA is also able to bind to several host receptors such as loricrin and CK10, supporting the adherence of *S. aureus* to desquamated nasal epithelial cells in humans and colonization in animal experiments [46]. Only a few LA-MRSA harbored *sdrC-D* genes even though *sdrE* gene was identified in all strains. The *sdrC-D* genes are related to adhesion to human epithelial cells; however, the *sdrE* gene is recognized as an inhibitor of both classical and alternative complement pathways [46,47].

Altogether, it can be summarized that all LA-MRSA isolates in our study pose virulence genes that can potentiate intercellular adhesion and colonization in human nostrils, facilitate biofilm formation, and promote a wide spectrum of staphylococcal infections. These findings support the conclusion that LA-MRSA can serve as an invasive pathogen threatening human health, especially in patients with immunocompromised or underlying medical conditions [5].

*In-silico* prediction of antimicrobial resistance gene-associated mobile genetic elements revealed two unique multi-resistant plasmids on draft genomes of LA-MRSA strains BA3.1 and J101.2; nevertheless, previous studies have reported the emergence of MRSA possessing multi-resistance gene clusters either on chromosome or plasmid. To illustrate this, the co-existence of numerous antimicrobial resistance genes on the LA-MRSA genome J101.2, including *aadD*, *blaZ*, *lnu*(B), and *Isa*(E) genes, had been detected earlier on a chromosome of MRSA ST9 isolated from frozen food in China [48]. Additionally, a multi-resistance gene cluster on a plasmid was identified in MRSA ST9 from a Chinese pig [49]. Apart from the aminoglycosides, beta-lactams, lincosamides, and PLS_A_ resistance genes, the tetracycline resistance gene *tet*(L) and MLS_B_ resistance *erm*(B) were co-localized on this plasmid, which were also found on our LA-MRSA strain BA3.1. The co-carriage of diverse antimicrobial resistance genes on the plasmids demonstrated in our study raises an awareness of significant risk related to antimicrobial resistance gene transfer [49].

We have, however, encountered some challenges in using short-read sequencing technologies. First, we could not access whole sequences of SCC*mec*CI elements carried by our LA-MRSA CC398; therefore; we cannot clarify whether the additional *ccr* gene complex was a result of two discrete integrated SCC*mec* elements [50]. Another limitation is that we were not able to visualize the genomic organization of multi-resistance gene clusters located on putative plasmid contigs. This would highlight that several antimicrobial resistance genes and other resistance genes can be horizontally transferred in a single event. To tackle these problems, the so-called hybrid genome assembly produced from long-read and short-read sequencing technologies will be implemented in order to obtain the reconstruction of a complete bacterial chromosome, SCC*mec* cassette, or plasmid in our future strategies.

## 4. Materials and Methods

### 4.1. LA-MRSA Isolates

A total of 16 representative isolates of LA-MRSA, which were previously characterized using molecular typing methods in 2019, were selected for WGS analyses in this study [2]. Nasal swabs were obtained from healthy swine farmers (*n* = 3) and live pigs (*n* = 13) in 11 swine farms located in the central region of Thailand, designated as Farm 1–Farm 11, 2015–2017 [2]. Bacterial stock was kept in tryptic soy broth (BD Difco, Franklin Lakes, NJ, USA) with 30% glycerol at −80 °C before recovery on 5% sheep blood agar for further processes.

### 4.2. DNA Extraction

The heat-killed bacterial solution of all representative isolates was transported to the Hokkaido University International Institute for Zoonosis Control, Japan, for DNA extraction. The genomic DNA (gDNA) was extracted by the bead-beating technique described previously [24]. Further, the concentration and quality of DNA were assessed using Qubit 3.0 Fluorometer and NanoDrop One Spectrophotometer (Thermo Fisher Scientific, Waltham, MA, USA).

### 4.3. Library Preparation, WGS and Genome Assembly

Paired-end libraries were prepared using the Nextera XT DNA Library Preparation Kit (Illumina Inc., San Diego, CA, USA). The concentration of each extracted gDNA sample was diluted to 0.2 ng/µL and subsequently sequenced on an Illumina MiSeq with 2 × 300 bp reads. The generated raw reads were quantified using FastQC v0.11.9 [51]. Then, the low-quality reads with a Phred quality score <20 were filtered out by Trim Reads Tool from CLC Genomics Workbench v22.0.1 (Qiagen, Hilden, Germany). The trimmed reads were assembled into contigs using SPAdes v3.15.4 on the Galaxy platform [52,53]. The assembly statistics were evaluated using QUAST v5.2.0 [54]. An N50 value of at least 50,000 was required for downstream analyses [55].

### 4.4. Genomic Characterization and In-Silico Identification of Antimicrobial Resistance, Stress, and Virulence Genes

*In-silico* MLST was performed to assign a sequence type to each LA-MRSA isolate using MLST v2.0 [56]. SCCmecFinder v1.2 with a minimum gene coverage of 80% and minimum identity cut-off of 90% was used to determine SCC*mec* types; spaTyper v1.0 was applied to identify *spa* types [57,58]. Acquired antimicrobial resistance genes, mutations in genes associated with antimicrobial resistance, and stress genes were *in-silico* screened using NCBI AMRFinderPlus v3.10.42 [59]. VFDB was primarily used for *in-silico* detection of virulence genes on the BV-BRC platform v3.28.5 [60,61]; however, the virulence genes that were not included in VFDB were additionally predicted by AMRFinderPlus. The minimum length and percentage identity of these two bioinformatic tools were adjusted to 80% and 90%, respectively, to determine the absence/presence of a particular gene.

### 4.5. Core Genome Alignment and Phylogenetic Tree Reconstruction

The trimmed paired-end reads of each LA-MRSA were aligned against the LA-MRSA ST398 reference genome (strain S0385; GenBank accession no. AM990992) using Snippy pipeline v4.6.0. Snippy-core v4.6.0 was used for SNP calling from core-genome alignment [62]. Gubbins was run to eliminate the polymorphic sites of recombination in the alignment [63]. Further, the number of pair-wise SNP distances was computed using Snp-Dists v0.8.2 [64]. The phylogenetic tree of LA-MRSA CC398 based on SNPs in the core genome was reconstructed on MEGA v11 [65]. The maximum-likelihood inference, together with Kimura’s two-parameter substitution model (K2P), was utilized to infer the evolutionary tree [66]. The bootstrap support values of 1000 replicates were calculated to assess the robustness of each node of the resulting tree. The final phylogenetic tree was visualized and integrated with a set of metadata using iTOL v6.8 [67].

## 5. Conclusions

Our study demonstrates how to apply WGS technologies for the epidemiological investigation of zoonotic transmission occupationally caused by LA-MRSA CC398 in Thailand. The WGS analyses with a high-resolution genomic approach also reveal genetic recombination through the evolutionary process, influenced by the introduction of an exotic strain of LA-MRSA CC398. Diverse antimicrobial resistance-related genes are widespread in our LA-MRSA isolates. Cross-resistance genes emphasize the judicious usage of antimicrobials in livestock production. The co-existence of several antimicrobial resistance genes on plasmids and the virulence gene repertoire reflects the robustness of biosecurity-associated strategies to confine horizontal gene transfer among bacterial communities outside agricultural areas as well as to reduce the risk of transmission at pre-harvest. These also accentuate the primary role of the One Health approach, collaboratively addressing antimicrobial resistance issues.

## Figures and Tables

**Table 1:** WGS-based characteristics, including *ccr* gene complex(es) and *mec* complex class identified in each representative of LA-MRSA isolates.

Strain Name	Farm	Location	ST	CC	SCC*mec* Type	*spa* Type	*mec* Complex Class	*ccr* Gene Complex			
								Type	*ccrA1*	*ccrB1*	*ccrC1*
Q10.1	6	PB	9	9	IX	t337	C2	1	+	+	-
Y1.3	8	PB	4576	9	IX	t337	C2	1	+	+	-
BA3.1	2	RB	9	9	IX	t337	C2	1	+	+	-
J101.2	11	PB	398	398	CI	t034	C2	NT	+	+	+
L3.1	1	NP	398	398	CI	t034	C2	NT	+	+	+
L43.2	1	NP	398	398	CI	t034	C2	NT	+	+	+
Z19.1	1	NP	398	398	CI	t034	C2	NT	+	+	+
AA3.1	4	SB	398	398	V	t034	C2	5	-	-	+
M3.1	3	SB	398	398	V	t034	C2	5	-	-	+
M31.1	3	SB	398	398	V	t034	C2	5	-	-	+
Y1.2	8	PB	398	398	V	t034	C2	5	-	-	+
S2.1	7	PB	398	398	V	t034	C2	5	-	-	+
H49.1	10	NR	398	398	V	t034	C2	5	-	-	+
G2.1	9	NR	398	398	V	t034	C2	5	-	-	+
D16.1	5	PB	398	398	V	t034	C2	5	-	-	+
X1.1	7	PB	398	398	V	t034	C2	5	-	-	+

PB: Prachin Buri, RB: Ratchaburi, NP: Nakhon Pathom, SB: Suphanburi, NR: Nakhon Ratchasima, NT: non-typable, +: detected, -: not detected.

**Table 2:** Distribution of 32 antimicrobial resistance-associated genes within the 16 LA-MRSA CC9 and CC398. Each gene was classified into 14 antimicrobial groups in accordance with their phenotypes. The grey color indicates the presence of genes or mutations in each isolate predicted by the AMRFinderPlus database.

Strain Name		Q10.1	Y1.3	BA3.1	J101.2	L3.1	L43.2	Z19.1	AA3.1	M3.1	M31.1	Y1.2	S2.1	H49.1	G2.1	D16.1	X1.1
Origin		Pig	Pig	Pig	Human	Pig	Human	Pig	Pig	Pig	Human	Pig	Pig	Pig	Pig	Pig	Pig
Farm		6	8	2	11	1	1	1	4	3	3	8	7	10	9	5	7
Location		PB	PB	RB	PB	NP	NP	NP	SB	SB	SB	PB	PB	NR	NR	PB	PB
ST		9	4576	9	398	398	398	398	398	398	398	398	398	398	398	398	398
CC		9	9	9	398	398	398	398	398	398	398	398	398	398	398	398	398
SCC*mec* type		IX	IX	IX	CI	CI	CI	CI	V	V	V	V	V	V	V	V	V
*spa* type		t337	t337	t337	t034	t034	t034	t034	t034	t034	t034	t034	t034	t034	t034	t034	t034
AMGs	*aac(6′)-Ie/aph(2″)-Ia*																
	*aadD1*																
	*ant(6)-Ia*																
	*ant(9)-Ia*																
	*spw*																
	*str*																
BLs	*blaPC1*																
	*blaZ*																
	*mecA*																
FQs	Mutation of *gyrA* S84L																
	Mutation of *parC* E84G																
	Mutation of *parC* S80F																
	Mutation of *parC* S80Y																
FOS	*fosB*																
	Mutation of *glpT* A100V																
	Mutation of *glpT* F3I																
	Mutation of *murA* D278E																
	Mutation of *murA* E291D																
GPAs	*vanA*																
LINs	*lnu*(B)																
PLS_A_	*lsa*(E)																
	*vga*(A)																
	*vga*(A)-LC																
MLS_B_	*erm*(A)																
	*erm*(B)																
	*erm*(C)																
PHEs	*catA*																
	*fexA*																
PhLOPS_A_	*cfr*																
RIF	Mutation of *rpoB*																
TCs	*tet*(38)																
	*tet*(K)																
	*tet*(L)																
	*tet*(M)																
TMP	*dfrG*																
MATE transporter	*mepA*																

AMGs: aminoglycosides, BLs: beta-lactams, FQs: fluoroquinolones, FOS: fosfomycin, GPAs: glycopeptide antibiotics, LINs: lincosamides, PLS_A_: lincosamides-pleuromutilins-streptogramin A compounds, MLS_B_: macrolide-lincosamide-streptogramin B compounds, PHEs: phenicols, PhLOPS_A_: phenicols-oxazolidinones-lincosamides-pleuromutilins-streptogramin A compounds, RIF: rifampicin, TCs: tetracyclines, TMP: trimethoprim, MATE: multidrug and toxic compound extrusion.

**Table 3:** Distribution of 76 virulence determinants among the 16 LA-MRSA CC9 and CC398. The grey color indicates the presence of genes harbored by each strain.

Strain Name		Q10.1	Y1.3	BA3.1	J101.2	L3.1	L43.2	Z19.1	AA3.1	M3.1	M31.1	Y1.2	S2.1	H49.1	G2.1	D16.1	X1.1
Origin		Pig	Pig	Pig	Human	Pig	Human	Pig	Pig	Pig	Human	Pig	Pig	Pig	Pig	Pig	Pig
Farm		6	8	2	11	1	1	1	4	3	3	8	7	10	9	5	7
Location		PB	PB	RB	PB	NP	NP	NP	SB	SB	SB	PB	PB	NR	NR	PB	PB
ST		9	4576	9	398	398	398	398	398	398	398	398	398	398	398	398	398
CC		9	9	9	398	398	398	398	398	398	398	398	398	398	398	398	398
SCC*mec* type		IX	IX	IX	CI	CI	CI	CI	V	V	V	V	V	V	V	V	V
*spa* type		t337	t337	t337	t034	t034	t034	t034	t034	t034	t034	t034	t034	t034	t034	t034	t034
Virulence genes related to adherence	*clfA*																
	*clfB*																
	*cna*																
	*ebp*																
	*icaA*																
	*icaB*																
	*icaC*																
	*icaD*																
	*icaR*																
	*map*																
	*sdrC*																
	*sdrD*																
	*sdrE*																
Virulence genes related to exoenzymes	*adsA*																
	*aur*																
	*Coa*																
	*geh*																
	*hysA*																
	*Lip*																
	sak *																
	*sspA*																
	*sspB*																
	*sspC*																
Virulence genes related to host immune evasion	*cap8A*																
	*cap8B*																
	*cap8C*																
	*cap8D*																
	*cap8E*																
	*cap8F*																
	*cap8G*																
	*cap8L*																
	*cap8M*																
	*cap8N*																
	*cap8O*																
	*cap8P*																
	*chp*																
	scn *																
	*sbi*																
	*spa*																
Virulence genes related to iron uptake and metabolism	*isdA*																
	*isdB*																
	*isdC*																
	*isdD*																
	*isdE*																
	*isdF*																
	*isdG*																
	*srtB*																
Virulence genes related to toxins and type IV secretion	*esaA*																
	*esaB*																
	*essA*																
	*essB*																
	*essC*																
	*esxA*																
	*esxB*																
	*esxC*																
	*hla*																
	*hlb*																
	*hld*																
	*hlgA*																
	*hlgB*																
	*hlgC*																
	*lukF-PV*																
	*lukS-PV*																
	sea *																
	sep *																
	sei *																
	sel26*																
	sel27 *																
	sel28 *																
	selx *																
	sem *																
	sen *																
	seo *																
	seu *																
	sey *																
	*tsst-1*																

* Virulence genes additionally predicted by the AMRFinderPlus.

**Table 4:** Distribution of mobile genetic elements with their associated antimicrobial resistance genes carried by the 16 LA-MRSA CC9 and CC398.

Strain Name	Origin	Farm	Location	ST	CC	SCC*mec* Type	*spa* Type	ARG-Associated Plasmid Replicon and Transposon	Insertion Sequence
Q10.1	Pig	6	PB	9	9	IX	t337	rep5d-*vga*(A)-LC	-
								rep7a-*str*-*cat*(pC221)	
								rep13-*qacG*	
								rep21	
								repUS43-*mecA*-*tet*(M)	
Y1.3	Pig	8	PB	4576	9	IX	t337	rep5d-*vga*(A)-LC	-
								rep7a-*str*	
								rep13-*qacG*	
								rep21	
								repUS43-*mecA*-*tet*(M)	
BA3.1	Pig	2	RB	9	9	IX	t337	rep7a-*str*	-
								rep10b-*vga*(A)-LC	
								repUS18-*aadD*-*erm*(B)-*tet*(L)	
								repUS43-*mecA*-*tet*(M)	
								Tn*558*	
J101.2	Human	11	PB	398	398	CI	t034	rep7a-*tet*(K)	*lS*256
								rep22-*aadD*-*ant(6)-la*-blaZ *-*lnu*(B)-*lsa*(E)	
								repUS43-*tet*(M)	
								Tn*551*-*erm*(B)	
								Tn*554*-*ant(9)-la*-*erm*(A)	
L3.1	Pig	1	NP	398	398	CI	t034	rep7a-*tet*(K)	-
								rep22-*aadD*	
								repUS43-*tet*(M)	
								Tn551-*erm*(B)	
								Tn*554*-*ant(9)-la*-*erm*(A)	
L43.2	Human	1	NP	398	398	CI	t034	rep7a-*tet*(K)	*lS*Sau8
								rep22-*aadD*	
								repUS43-*tet*(M)	
								Tn*551*-*erm*(B)	
								Tn*554*-*ant(9)-la*-*erm*(A)	
Z19.1	Pig	1	NP	398	398	CI	t034	rep7a-*tet*(K)	-
								rep22-*aadD*	
								repUS43-*tet*(M)	
								Tn*551*-*erm*(B)	
								Tn*554*-*ant(9)-la*-*erm*(A)	
AA3.1	Pig	4	SB	398	398	V	t034	rep7a-*tet*(K)	-
								rep10-*erm*(C)	
								rep19b-*blaZ*	
								rep21	
								repUS43-*tet*(M)-Tn*6009*	
M3.1	Pig	3	SB	398	398	V	t034	rep7a	-
								rep10-*erm*(C)	
								rep19b	
								rep21	
								repUS43-*tet*(M)-Tn*6009*	
M31.1	Human	3	SB	398	398	V	t034	rep7a-*tet*(K)	-
								rep10-*erm*(C)	
								rep19b-*blaZ*	
								rep21	
								repUS43-*tet*(M)-Tn*6009*	
Y1.2	Pig	8	PB	398	398	V	t034	rep7a-*tet*(K)	-
								rep10	
								rep19b	
								repUS43-*tet*(M)-Tn*6009*	
S2.1	Pig	7	PB	398	398	V	t034	rep7a-*tet*(K)	-
								rep10-*erm*(C)	
								rep13-*qacG*	
								rep19b-*blaZ*	
								rep21	
								repUS43-*tet*(M)-Tn*6009*	
H49.1	Pig	10	NR	398	398	V	t034	rep7a-*tet*(K)	-
								rep10-*erm*(C)	
								rep19b-*blaZ*	
								repUS43-*tet*(M)-Tn*6009*	
G2.1	Pig	9	NR	398	398	V	t034	rep7a-*tet*(K)	-
								rep10-*erm*(C)	
								rep19b-*blaZ*	
								rep21	
								repUS43-*tet*(M)-Tn*6009*	
D16.1	Pig	5	PB	398	398	V	t034	rep7a-*tet*(K)	*lS*Sau8
								rep10-*erm*(C)	
								rep13	
								rep19b-*blaZ*	
								rep21	
								repUS43-*tet*(M)-Tn*6009*	
X1.1	Pig	7	PB	398	398	V	t034	rep7a-*tet*(K)	*lS*Sau8
								rep10-*erm*(C)	
								rep19b-*blaZ*	
								repUS43-*tet*(M)-Tn*6009*	

* This gene was not detected in the human strain J101.2 using the AMRFinderPlus tool. ARG: antimicrobial resistance gene, -: not detected.

## Data Availability

Data are contained within the article and Supplementary Materials.

## Supplementary Materials

The following supporting information can be downloaded at: https://www.mdpi.com/2079-6382/12/12/1745/s1. Table S1: pairwise SNP distance matrix of the 13 LA-MRSA CC398 isolates based on core-genome alignment; Table S2: distribution of five stress genes identified by the AMRFinderPlus database among the 16 LA-MRSA CC9 and CC398; Figure S1: map focusing on the central region of Thailand. Refs. [68,69,70,71] are cited in the Supplementary Materials.
