# Correction: Narongpun et al. Whole-Genome Investigation of Zoonotic Transmission of Livestock-Associated Methicillin-Resistant *Staphylococcus aureus* Clonal Complex 398 Isolated from Pigs and Humans in Thailand. *Antibiotics* 2023, *12*, 1745

**DOI:** 10.3390/antibiotics13080681

**Published:** 2024-07-23

**Authors:** Pawarut Narongpun, Pattrarat Chanchaithong, Junya Yamagishi, Jeewan Thapa, Chie Nakajima, Yasuhiko Suzuki

**Affiliations:** 1 Division of Bioresources, Hokkaido University International Institute for Zoonosis Control, Sapporo 001-0020, Japan; npawarut@czc.hokudai.ac.jp (P.N.); jeewan@czc.hokudai.ac.jp (J.T.); 2 Department of Veterinary Microbiology, Faculty of Veterinary Science, Chulalongkorn University, Bangkok 10330, Thailand; pattrarat.c@chula.ac.th; 3 Division of Collaboration and Education, Hokkaido University International Institute for Zoonosis Control, Sapporo 001-0020, Japan; junya@czc.hokudai.ac.jp; 4 International Collaboration Unit, Hokkaido University International Institute for Zoonosis Control, Sapporo 001-0020, Japan; 5 Institute for Vaccine Research and Development, Hokkaido University, Sapporo 001-0020, Japan

## Table 2 Needs to Be Changed

In the published publication [1], there was an error regarding Table 2. The published Table 2 was incomplete. The correct version of Table 2 should be the following. The authors state that the scientific conclusions are unaffected. This correction was approved by the Academic Editor. The original publication has also been updated. 

**Table 2:** Distribution of 32 antimicrobial resistance-associated genes within the 16 LA-MRSA CC9 and CC398. Each gene was classified into 14 antimicrobial groups in accordance with their phenotypes. The grey color indicates the presence of genes or mutations in each isolate predicted by the AMRFinderPlus database.

Strain Name		Q10.1	Y1.3	BA3.1	J101.2	L3.1	L43.2	Z19.1	AA3.1	M3.1	M31.1	Y1.2	S2.1	H49.1	G2.1	D16.1	X1.1
Origin		Pig	Pig	Pig	Human	Pig	Human	Pig	Pig	Pig	Human	Pig	Pig	Pig	Pig	Pig	Pig
Farm		6	8	2	11	1	1	1	4	3	3	8	7	10	9	5	7
Location		PB	PB	RB	PB	NP	NP	NP	SB	SB	SB	PB	PB	NR	NR	PB	PB
ST		9	4576	9	398	398	398	398	398	398	398	398	398	398	398	398	398
CC		9	9	9	398	398	398	398	398	398	398	398	398	398	398	398	398
SCC*mec* type		IX	IX	IX	CI	CI	CI	CI	V	V	V	V	V	V	V	V	V
*spa* type		t337	t337	t337	t034	t034	t034	t034	t034	t034	t034	t034	t034	t034	t034	t034	t034
AMGs	*aac(6′)-Ie/aph(2″)-Ia*																
	*aadD1*																
	*ant(6)-Ia*																
	*ant(9)-Ia*																
	*spw*																
	*str*																
BLs	*blaPC1*																
	*blaZ*																
	*mecA*																
FQs	Mutation of *gyrA* S84L																
	Mutation of *parC* E84G																
	Mutation of *parC* S80F																
	Mutation of *parC* S80Y																
FOS	*fosB*																
	Mutation of *glpT* A100V																
	Mutation of *glpT* F3I																
	Mutation of *murA* D278E																
	Mutation of *murA* E291D																
GPAs	*vanA*																
LINs	*lnu*(B)																
PLS_A_	*lsa*(E)																
	*vga*(A)																
	*vga*(A)-LC																
MLS_B_	*erm*(A)																
	*erm*(B)																
	*erm*(C)																
PHEs	*catA*																
	*fexA*																
PhLOPS_A_	*cfr*																
RIF	Mutation of *rpoB*																
TCs	*tet*(38)																
	*tet*(K)																
	*tet*(L)																
	*tet*(M)																
TMP	*dfrG*																
MATE transporter	*mepA*																

AMGs: aminoglycosides, BLs: beta-lactams, FQs: fluoroquinolones, FOS: fosfomycin, GPAs: glycopeptide antibiotics, LINs: lincosamides, PLS_A_: lincosamides-pleuromutilins-streptogramin A compounds, MLS_B_: macrolide-lincosamide-streptogramin B compounds, PHEs: phenicols, PhLOPS_A_: phenicols-oxazolidinones-lincosamides-pleuromutilins-streptogramin A compounds, RIF: rifampicin, TCs: tetracyclines, TMP: trimethoprim, MATE: multidrug and toxic compound extrusion.
