# Supplementary material for: Whole-Genome Investigation of Zoonotic Transmission of Livestock-Associated Methicillin-Resistant Staphylococcus aureus Clonal Complex 398 Isolated from Pigs and Humans in Thailand
Source: Antibiotics (Basel). 2023 Dec 16;12(12):1745. doi: 10.3390/antibiotics12121745 (PMC10741195; doi:10.3390/antibiotics12121745)
Supplement: Supplementary file 1 [file antibiotics-12-01745-s001.zip › antibiotics-2726666-supplementary.pdf]

antibiotics

MDPI

Supplementary Materials

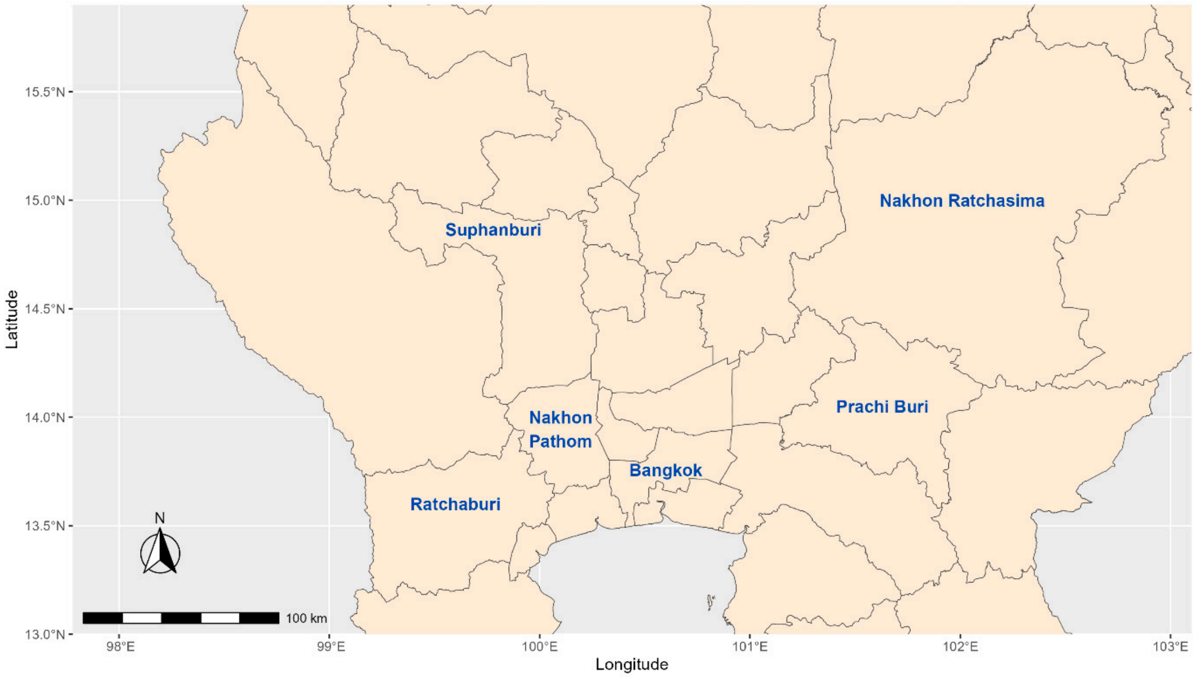

Figure S1. Map focusing on the central region of Thailand. It presents the location of 6 cities, 5 of which are sources of the 16 representative isolates of LA-MRSA CC9 and CC398. The capital city of Bangkok is used as a landmark. This map was created by several packages, including ggplot2 [68], raster [69], sf [70] and tidyverse [71], on RStudio v2023.06.1+524 (https://rstudio.com/, accessed on 18 July 2023). Geometric data of province boundaries were downloaded from the Database of Global Administrative Areas v4.1 (https://gadm.org/, accessed on 18 July 2023).

Table S1. Pairwise SNP distance matrix of the 13 LA-MRSA CC398 isolates based on core genome alignment. The minimum pairwise SNP distances between porcine and human isolates were 10 and 3 in Farm 1 and Farm 3, respectively, verifying the pig-to-human transmission caused by LA-MRSA CC398.

Pairwise SNP distance matrix of the 13 LA-MRSA CC398 isolates			L3.1	L43.2	Z19.1	M3.1	M31.1	AA3.1	D16.1	S2.1	X1.1	Y1.2	G2.1	H49.1	J101.2
			Farm 1	Farm 1	Farm 1	Farm 3	Farm 3	Farm 4	Farm 5	Farm 7	Farm 7	Farm 8	Farm 9	Farm 10	Farm 11
			Pig	Human	Pig	Pig	Human	Pig	Pig	Pig	Pig	Pig	Pig	Pig	Human
**L3.1**	**Farm 1**	**Pig**	0	**11**	13	503	506	505	506	500	508	476	501	491	239
**L43.2**	**Farm 1**	**Human**	11	0	10	503	506	505	506	500	508	476	501	491	238
**Z19.1**	**Farm 1**	**Pig**	**13**	**10**	0	504	507	506	507	501	509	477	502	492	240
**M3.1**	**Farm 3**	**Pig**	503	503	504	0	**3**	**2**	51	45	53	37	48	40	557
**M31.1**	**Farm 3**	**Human**	506	506	507	3	0	**3**	38	46	54	38	49	41	560
**AA3.1**	**Farm 4**	**Pig**	505	505	506	2	3	0	51	45	53	37	48	40	559
**D16.1**	**Farm 5**	**Pig**	506	506	507	51	52	51	0	38	14	29	23	21	560
**S2.1**	**Farm 7**	**Pig**	500	500	501	45	46	45	38	0	40	24	35	27	554
**X1.1**	**Farm 7**	**Pig**	508	508	509	53	54	53	14	40	0	30	25	23	562
**Y1.2**	**Farm 8**	**Pig**	476	476	477	37	37	37	29	24	30	0	26	20	515
**G2.1**	**Farm 9**	**Pig**	501	501	502	48	49	48	23	35	25	26	0	18	555
**H49.1**	**Farm 10**	**Pig**	491	491	492	40	41	40	21	27	23	20	18	0	545
**J101.2**	**Farm 11**	**Human**	239	238	240	557	560	559	560	554	562	515	555	545	0

Table S2. Distribution of five stress genes identified by the AMRFinderPlus database among the 16 LA-MRSA CC9 and CC398.

Strain name	Origin	Farm	Location	Sequence type	Clonal complex	SCC*mec* type	*spa* type	Arsenite efflux transporter membrane subunit	Thioredoxin-dependent arsenate reductase	Multidrug efflux MFS transporter	Multi-copper oxidase	Quaternary ammonium compound efflux SMR transporter
								arsB	*arsC*	*lmrS*	*mco*	*qacG*
Q10.1	Pig	6	Prachin Buri	9	9	IX	t337					
Y1.3	Pig	8	Prachin Buri	4576	9	IX	t337					
BA3.1	Pig	2	Ratchaburi	9	9	IX	t337					
J101.2	Human	11	Prachin Buri	398	398	CI	t034					
L3.1	Pig	1	Nakhon Pathom	398	398	CI	t034					
L43.2	Human	1	Nakhon Pathom	398	398	CI	t034					
Z19.1	Pig	1	Nakhon Pathom	398	398	CI	t034					
AA3.1	Pig	4	Suphanburi	398	398	V	t034					
M3.1	Pig	3	Suphanburi	398	398	V	t034					
M31.1	Human	3	Suphanburi	398	398	V	t034					
Y1.2	Pig	8	Prachin Buri	398	398	V	t034					
S2.1	Pig	7	Prachin Buri	398	398	V	t034					
H49.1	Pig	10	Nakhon Ratchasima	398	398	V	t034					
G2.1	Pig	9	Nakhon Ratchasima	398	398	V	t034					
D16.1	Pig	5	Prachin Buri	398	398	V	t034					
X1.1	Pig	7	Prachin Buri	398	398	V	t034					

Grey color indicates the presence of genes harbored by each isolate. MFS: the major facilitator superfamily, SMR: small multidrug resistance.
